# New Genetic Loci Associated with Preharvest Sprouting and Its Evaluation Based on the Model Equation in Rice

**DOI:** 10.3389/fpls.2017.01393

**Published:** 2017-08-08

**Authors:** Gi-An Lee, Young-Ah Jeon, Ho-Sun Lee, Do Yoon Hyun, Jung-Ro Lee, Myung-Chul Lee, Sok-Young Lee, Kyung-Ho Ma, Hee-Jong Koh

**Affiliations:** ^1^National Agrobiodiversity Center, National Institute of Agricultural Sciences Jeonju, South Korea; ^2^Department of Plant Science, Plant Genomics and Breeding Institute, and Research Institute of Agriculture and Life Sciences, Seoul National University Seoul, South Korea; ^3^International Technology Cooperation Center Jeonju, South Korea

**Keywords:** rice, genetic resources, preharvest sprouting (PHS), dormancy, regression model

## Abstract

Preharvest sprouting (PHS) in rice panicles is an important quantitative trait that causes both yield losses and the deterioration of grain quality under unpredictable moisture conditions at the ripening stage. However, the molecular mechanism underlying PHS has not yet been elucidated. Here, we explored the genetic loci associated with PHS in rice and formulated a model regression equation for rapid screening for use in breeding programs. After re-sequencing 21 representative accessions for PHS and performing enrichment analysis, we found that approximately 20,000 SNPs revealed distinct allelic distributions between PHS resistant and susceptible accessions. Of these, 39 candidate SNP loci were selected, including previously reported QTLs. We analyzed the genotypes of 144 rice accessions to determine the association between PHS and the 39 candidate SNP loci, 10 of which were identified as significantly affecting PHS based on allele type. Based on the allele types of the SNP loci, we constructed a regression equation for evaluating PHS, accounting for an *R^2^* value of 0.401 in *japonica* rice. We validated this equation using additional accessions, which exhibited a significant *R^2^* value of 0.430 between the predicted values and actual measurements. The newly detected SNP loci and the model equation could facilitate marker-assisted selection to predict PHS in rice germplasm and breeding lines.

## Introduction

Rice, possessing a relatively small genome size of approximately 370 Mb, is widely used in genomic studies as a model cereal crop plant. Identifying important genes in rice is an excellent approach for finding functional genes in grass-family crops such as wheat (hexaploid) and barley by comparative genomics (He et al., 2007; Liu et al., 2012). Next generation sequencing (NGS) was recently used to detect candidate genes associated with complex traits in diverse genetic backgrounds, and genome-wide associated studies based on haplotype maps have revealed several candidate genes for various agronomic traits in rice (Huang et al., 2010).

Preharvest sprouting (PHS) in rice panicles is caused by the breakage of adequate seed dormancy, while, under normal seed dormancy, the maturation status of the seed is arrested for various periods of time to allow it to germinate under favorable conditions (Gubler et al., 2005). PHS is an important trait in cereal crops, as it reduces grain yields and grain quality under unpredictable moisture conditions (Bewley and Black, 1982; Bailey et al., 1999; Li et al., 2004). Therefore, improving PHS resistance is a major breeding target for cereal crops worldwide (Zhang et al., 2014), and understanding of the genetic basis of seed dormancy and its breakage is important for regulating PHS in cereal crops.

The major genes associated with seed dormancy and germination reported to date are related to the biosynthesis, catabolism, perception, and signal transduction of abscisic acid (ABA), revealing its central role in controlling seed dormancy. Arabidopsis *AtABI3* plays a role in the initial ABA-dependent check point for seed dormancy, and its orthologous genes *ZmVP-1* in maize, *OsVP1* in rice and *TaVp-1* in wheat are global regulators of seed maturation in these crops (Hattori et al., 1994; Nakamura and Toyama, 2001; Lopez-Molina et al., 2002; De Laethauwer et al., 2012; Huang et al., 2012).

In rice, *Sdr4* is positively regulated by *OsVP1*, which acts as an intermediate regulator in the genetic regulation of seed dormancy (Sugimoto et al., 2010), and Zhang et al. (2014) isolated *TaSdr*, which is associated with tolerance to PHS, and developed a functional marker in wheat in a comparative study based on rice *Sdr4* (Zhang et al., 2014). Mutations in genes that participate in the biosynthesis of the carotenoid precursors of ABA cause PHS in rice (Fang et al., 2008). The dormancy QTL *qSD12*, which was delimited to a 75 kb region of chromosome 12, includes two candidate underlying genes: *PIL5* (phytochrome-interacting factor3-like 5) and *bHLH* (basic helix-loop-helix) (Gu et al., 2010). *qSD7-1/qPC7* (*Rc* locus) is a pleiotropic gene that promotes the expression of key genes for ABA biosynthesis and seed pigmentation (Gu et al., 2011). Although several QTLs associated with seed dormancy and PHS caused by the breakage of adequate dormancy have been reported (Dong et al., 2003; Gao et al., 2008; Hori et al., 2010) and both fine mapping analysis based on QTLs and mutant studies have been conducted to unveil the genetic basis of seed dormancy and PHS (Dong et al., 2003; Fang et al., 2008; Gao et al., 2008; Gu et al., 2010, 2011; Hori et al., 2010; Sugimoto et al., 2010), the molecular mechanism of dormancy release and PHS has been unclear. Therefore, many more genes and alleles associated with seed dormancy and PHS must be identified to help breeders overcome the problem of PHS in cereal crops under unpredictable climate conditions. Magwa et al. (2016) reported genome-wide 16 loci significantly associated with seed germination in diverse rice germplasm by association mapping.

We previously analyzed the variation in PHS and seed germination among various rice genetic resources to increase the number of available alleles for PHS, as most studies of PHS and seed dormancy have been performed using limited resources and alleles from some well-known accessions (Lee et al., 2016); In this study, wide variations in PHS degree were discovered and the increase in germination of detached seeds from the panicle was detected among diverse rice genetic resources. Interestingly, PHS-susceptible accessions maintained higher or similar ABA levels compared with PHS-resistant accessions, suggesting that the key factors for seed dormancy and its breakage could be ABA perception and signal transduction.

The seed germination test after cultivation in Korean middle and southern region for 2 years revealed similar variation of seed germinability (data not shown), and these reflected that some genetic factors artificially selected during rice domestication might affect the diversity of seed germination besides environmental effects. In the current study, we searched for SNPs showing distinct allelic distribution between PHS resistant and susceptible accessions based on genome re-sequencing of representative accessions. We then genotyped the putative PHS-associated SNPs in rice germplasm and constructed a regression equation for the quantitative trait PHS, which could facilitate breeding for PHS resistance in rice.

## Materials and Methods

### Characterization of PHS and GI

The flowering date were tagged by panicles of each accession, and seeds and panicles having same flowering date (**Table 1** and Supplementary Tables 1, 7) were harvested at 42 days after flowering (DAF) in the various rice accessions. The moisture content was adjusted by drying at 15°C (RH 10%) during a 7 days period, as previously described (Lee et al., 2016). The susceptibility for PHS (PHS value) was surveyed using three panicles per each accession, which were incubated at 25°C (RH 100%) for 7 days, after which the number of germinated seeds on each panicle was recorded and expressed as a percentage of the total grain number per panicle. The lower the PHS value, the lower the susceptibility to PHS and the higher the resistance. Seed germination at harvesting time (GHT) was determined by threshing the panicles and planting three replications of 50 seeds (fruits in hulls) onto moistened Whatman filter paper (10 mL of distilled water) in Petri dishes. The seeds were incubated at 25°C (RH 100%) based on International Seed Testing Association (ISTA) guidelines, and germinated seeds (radicle and coleoptile emerged from the hull) were counted daily for a period of 10 days. The cumulative number of germinated seeds was expressed as the percentage of seeds planted. Germination index (GI) was calculated as described by Basra et al. (2005).

**Table 1:** Accessions used for the detection of divergent SNPs based on the PHS trait using re-sequencing and enrichment analysis.

Ecotype	Group	N	Accessions
*japonica*	PHS susceptible	8	Hyangjeomdo_IT204516(CHN), Janggyeong 1_IT204522(CHN), white rice_IT246754(KGZ), GIZA 159_IT001447(EGY), Gopum_Var-1(KOR), Migwang_Var-2(KOR), Woonbong 40_Var-3(KOR), Hwayoung_Var-4(KOR)
	PHS resistant	6	Share-192-1-B_IT213660(KOR), Chungseungjaerae_IT214294(JPN), Koshihikari_IT226904(JPN), Xiaozhanjiangmidao_IT225135(CHN), Jowoon_Var-5(KOR), Joongsaenggold_Var-6(KOR)
*indica*	PHS susceptible	3	CHACHME_IT679(TWN), Tianshangu_IT223668(CHN), KULU_IT2805(AUS)
	PHS resistant	4	Aswina_IT251140(BGD), KELEE_IT259863(BGD), DaccA14_IT259940(IND), Dasan_Var-7(KOR)

### DNA Re-sequencing and Detection of Divergent SNPs by PHS Group

Twenty-one samples (*japonica*, 14; *indica/tongil*, 7; **Table 1**), revealing similar flowering times (early August), were selected as representatives by PHS resistance (PHS resistant representatives: PHS < 20%, PHS susceptible representatives: PHS > 40%) at 42 DAF among the diverse rice genetic resources (Lee et al., 2016) in that environmental noises might reduce the resolution for the detection of PHS associated genetic loci in the field condition test. The samples were re-sequenced using the Illumina HiSeq 2500 platform to identify genome-wide variations and to detect genetic signals associated with PHS.

Genomic DNA was extracted from the 21 rice samples using a Gentra Puregene Cell Kit for Plants (Qiagen, Hilden, Germany). Library construction and sequencing data collection were conducted using Illumina’s official protocol with a 101 bp paired-end read length. Trimmomatic-0.33 (Bolger et al., 2014) was used to remove adapters and low-quality reads. The adapter-free trimmed reads were mapped against the reference genome of *Oryza sativa* L. (IRGSP-1.0.27) using Bowtie2 with default parameters (Langmead and Salzberg, 2012). The mapped reads were assigned to read groups and sorted using Picard version 1.138.^1^ Picard was also used to remove potential PCR duplicates and to repair mismatches between each read and its mate pair. For the subsequent local realignment, base quality recalibration, and variant calling steps, Genome Analysis Toolkit (GATK) version 3.4.46 (McKenna et al., 2010) was used. Local realignment of reads was carried out to correct misalignments caused by the presence of InDels. Base quality recalibration was performed to compensate for base quality errors from empirical measurements. For variant calling, arguments including “UnifiedGenotyper” and “SelectVariants” were used. Finally, the identified variants were filtered using the “VariantFiltration” argument based on the following criteria: (1) Reads with a mapping quality of zero, MQ0 higher than 4, and MQ0/(1.0^∗^DP) higher than 0.1, where DP is the unfiltered read depth, (2) FS higher than 200 to reduce false positives, and (3) Phred-scaled quality score lower than 30.

To identify specific SNPs in the PHS groups (PHS resistant and susceptible group), Fisher’s exact test implemented in SNPSift was conducted to analyze the resulting genotype count data (Cingolani et al., 2012) contrary to the linear or mixed model generally used for genome-wide association studies (GWAS) (Korte and Farlow, 2013). Information about PHS-specific groups and the genotype data assigned to the dominant and recessive models of the 2 × 2 contingency tables were used in this statistical test. To minimize the rate of false positives, multiple Bonferroni correction was conducted. SNPs were considered significant when the *p*-value of the total number of tests was below 0.05. Significantly enriched SNPs were annotated with SNPeff.

### Genotyping of Candidate SNPs in Diverse Rice Germplasm

Thirty-nine candidate SNP loci (for genotyping diverse rice genetic resources) were selected, including SNPs in germination-related genes with hormonal action, such as ABA perception and signal transduction, as well as SNPs in genes expressed specifically in florets and grains (Supplementary Table 8). A total of 144 accessions (80 *japonica* accessions and 64 *indica* accessions) showing PHS variations (Supplementary Table 7) were used to genotype the candidate SNPs, and an additional 56 *japonica* accessions (mainly Korean landraces) were used to validate the regression model.

Genotyping was performed using Fluidigm 192.24 Dynamic Array integrated fluid circuits (Fluidigm Incorporated, San Francisco, CA, United States). The IFC Controller utilizes pressure to control the valves in the chips and to the load samples and genotyping assay reagents into the reaction chambers. The EP-1 system was used as an endpoint image reader. Specific target amplification (STA) reactions were performed in a GeneAmp PCR System 9700 from Applied Biosystems.

For SNP genotyping, 5 μL of STA mix was prepared for each sample, containing 1 × Qiagen Multiplex PCR Master Mix (Qiagen, PN 206143) and 50–60 ng of genomic DNA. After diluting the STA mix, an allele-specific SNP genotyping assay was carried out with the SNPtype assays protocol in the Fluidigm 192.24 Dynamic Array IFC User’s Guide (PN 68000098 N1). After PCR amplification, the endpoint fluorescent image data were acquired on the EP-1 System. Data were analyzed using Fluidigm SNP Genotyping Analysis software to obtain genotype calls.

Since the *japonica* and *indica* ecotypes have different genetic constitutions, the accessions were separately analyzed based on ecotype (Morishima and Oka, 1981; Sun et al., 2002, 2015). Despite the scarcity of suitable molecular markers for seed germinability, some reported markers were available, such as InDel and SNP loci in *qLTG3-1* controlling low-temperature germinability in rice (Fujino et al., 2008; Fujino and Sekiguchi, 2011); these markers (and modified markers) were used to elaborate the regression equation for PHS.

### Data Analysis

One-way analysis of variance (ANOVA), Duncan’s multiple range test (DMRT), correlation analysis, and regression analysis were conducted using R software (ver. 3.2.3)^2^, and differences were considered significant when *p-value* < 0.05. The spatio-temporal expression patterns of genes in various tissues throughout plant growth were surveyed using the Rice Expression Profile Database (RiceXPro)^3^.

## Results

### Sequencing of Representative Rice Accessions to Detect Genome-Wide Variation

A total of 21 accessions with similar heading dates were sequenced using an Illumina HiSeq 2500 sequencer to identify polymorphisms between PHS resistant and susceptible accessions (**Table 1**). The genomes of these selected accessions were sequenced at an average of ∼25.7 × coverage, with an average of 9.59 Gb total bases (8.01–10.82) and 94.9 million reads (Supplementary Table 1). The sequences were aligned against the reference genome of *Oryza sativa* L. (IRGSP-1.0.27) using Bowtie2 with default parameters (Langmead and Salzberg, 2012). The alignment rate ranged from 92.8% (IT226904, Koshihikari) to 98.7% (IT 259863, KELEE), with a mean alignment rate of 96.8%. After filtering of potential PCR duplicates and correction of misalignments due to the presence of InDels, the genome-wide SNPs were detected using GATK (McKenna et al., 2010). To minimize the number of false-positive calls, we used several filtering steps before subjecting the candidate SNPs to further analyses based on the following: Phred-scaled quality score, mapping quality, quality depth, and Phred-scaled *p*-value. We ultimately acquired approximately 4.27 million genome-wide SNPs, with an average ratio of one SNP per 87 bp (Supplementary Table 2).

### Distribution of SNPs Showing Allelic Differentiation between PHS Resistant and Susceptible Accessions

We conducted enrichment analysis using Fisher’s exact test implemented in SNPSift (Cingolani et al., 2012) to detect allelic differentiation between PHS resistant and susceptible accessions and annotated significantly enriched SNPs in each PHS grou2.p using SNPef. Among the 4.27 million SNPs detected genome-wide, approximately 21,000 and 18,000 SNP loci showed distinguished allelic distribution between PHS resistant and susceptible accessions (21 accessions, including 14 *japonica* and 7 *indica/tongil*) based on dominant and recessive models (*p-value* < 0.05), respectively, and the common SNPs detected in both model were 16,753 loci (**Table 2** and Supplementary Tables 3, 4). Among the 14 *japonica* accessions, approximately 5,600 and 4,300 SNPs were detected based on dominant and recessive models, respectively, and the common SNPs detected in both models were 4,115 loci (**Table 2** and Supplementary Tables 5, 6).

**Table 2:** Summary of PHS-associated SNPs based on enrichment analysis.

Fisher’s exact test	No. of accessions	Total no. of detected SNPs (Common SNPs ¶)	No. of SNPs in genic reg. (Common SNPs ¶)
Dominant	21 accessions	21,032 (16,753)	4,119 (3,520)
model	14 *japonica* accessions	5,644 (4,115)	1,038 (844)
Recessive	21 accessions	17,988 (16,753)	3,707 (3,520)
model	14 *japonica* accessions	4,309 (4,115)	874 (844)

*¶Common SNPs: detected SNPs in both dominant and recessive model.*

Among the SNPs showing allelic differentiation between PHS resistant and susceptible accessions, the number of SNPs in genic regions was approximately 4,100 and 1,000 among the 21 total accessions (*japonica*, 14; *indica*, 6; *tongil*, 1) and the 14 *japonica* accessions, respectively (Supplementary Tables 3, 5), based on the dominant model and approximately 3,700 and 800 among the 21 and 14 accessions, respectively, based on the recessive model (Supplementary Tables 4, 6). Among the genic SNPs, intron variants were most prevalent among the 21 accessions (dominant model: 48.3% and recessive model: 48.0%), followed by exon variants (34.0 and 34.3%), 3′ UTR variants (11.4 and 11.3%), and 5′ UTR variants (6.4 and 6.4%). Among the 14 *japonica* accessions, exon variants were most prevalent (dominant model: 43.4% and recessive model: 44.6%), followed by intron variants (31.1 and 28.9%), 3′ UTR variants (16.5 and 16.9%), and 5′ UTR variants (9.1 and 9.5%).

### Chromosomal Distribution of SNPs in Different Alleles Based on PHS Group

**FIGURE 1:**
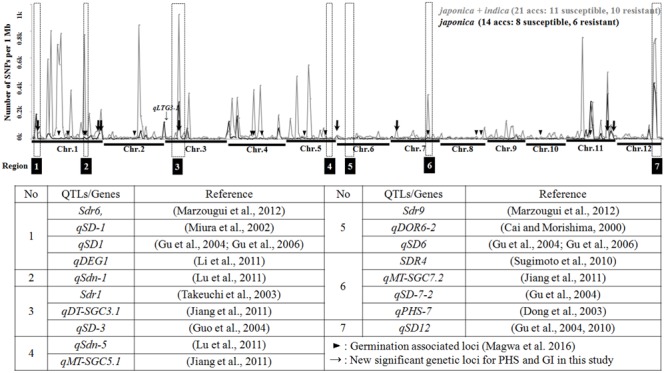
SNP Density plot of SNPs between PHS resistant and susceptible accessions and the locations of reported QTLs; SNP loci associated with PHS and GI in tested accessions are presented.

We constructed a density plot of SNPs in PHS resistant versus susceptible accessions using a 1 Mb bin size along the rice genome (**Figure 1**). Among the SNPs detected genome-wide, an average of 55 SNPs per bin revealed allelic differentiation between PHS resistant and susceptible accessions. Chromosome-1 has the highest number of SNPs, with an average of 142 per bin, followed by chromosome-11 (78 per bin) and chromosome-12 (69 per bin). Chromosome-8 has the fewest SNPs (12 per bin). Among the *japonica* accessions, only an average of 13 SNPs per bin revealed allelic differentiation between the PHS resistant and susceptible groups. Chromosome-11 has the highest number of SNPs, averaging 36 per bin, followed by chromosome-12 (34 per bin) and chromosome-4 (16 per bin). The fewest SNPs were found on chromosome-10 (three per bin). We compared the locations of the SNPs with publicly reported QTLs related to PHS and dormancy along the rice chromosome, finding that some chromosomal regions contain high numbers of these SNP loci. Region-1 (Chr.1: ∼1–5 Mb, 71 genic SNPs / 184 common SNPs) has a high density of SNPs overlapping with QTL *Sdr6* (Marzougui et al., 2012), *qSD-1* (Miura et al., 2002), *qSD1* (Gu et al., 2004, 2006) and *qDEG1* (Li et al., 2011). Region-2 (Chr.1: ∼31–33 Mb, 140 genic SNPs/787 common SNPs) contains the QTL *qSdn-1* (Lu et al., 2011), while Region-3 (Chr.3: ∼3–11 Mb) contains the QTLs *Sdr1* (Takeuchi et al., 2003), *qDT-SGC3.1* (Jiang et al., 2011) and *qSD-3* (Guo et al., 2004). Region-6 (Chr.7: ∼22–25 Mb, 91 genic SNPs / 787 common SNPs) harbors QTLs overlapping with *qSD-7-2* (Gu et al., 2004), *qMT-SGC7.2* (Jiang et al., 2011), *qPHS-7* (Dong et al., 2003) and fine-mapped *Sdr4* gene (Sugimoto et al., 2010). Region-7 (Chr.12: ∼21–27 Mb, 386 genic SNPs/1,391 common SNPs) harbors the QTL *qSD-12* (Gu et al., 2004) and fine mapped *qSD-12* gene (Gu et al., 2010). Although several studies reported QTLs in Region-4 (Chr.5: ∼24–28 Mb, 1 genic SNPs/5 common SNPs) and Region-5 (Chr.6: ∼6–12 Mb, 0 genic SNPs/4 common SNPs), we detected few SNPs in these regions in the current study. Other chromosomal regions revealing high density of SNPs without overlapping with reported QTLs included several germination associated loci by GWAS study (Magwa et al., 2016).

### Genotyping of Candidate SNPs and Their Association with PHS in Various Rice Germplasm

Among the approximately 20,000 SNP loci revealing distinct allelic distribution between PHS resistant and susceptible accessions, we genotyped 144 rice germplasm (80 *japonica* accessions and 64 *indica* accessions, Supplementary Table 7) using the 39 SNPs mainly located in genic or promoter region of candidate genes (Supplementary Table 8). We analyzed the differences in PHS and GI values according to genotype. Six and seven loci, including *qLTG3-1* (Fujino et al., 2008; Fujino and Sekiguchi, 2011) revealed significant differences in PHS and GI values according to allele type in 80 *japonica* accessions (*p-value <* 0.05 and *p-value <* 0.01), respectively (**Table 3**). Furthermore, the existence of an awn was also significantly associated with PHS and GI values. Three significant SNP loci were associated with PHS and GI values in 64 *indica* accessions. Specifically, among significant SNPs associated with PHS in *japonica* and *indica* rice, V2 is a significant variant in an upstream region of the rice homolog ortholog of *AtBG1*, which functions in ABA conjugation in Arabidopsis. V5 is a variant in the intron region of a *ROS* homeostasis gene, and the S4 locus is located in the upstream region of *OsVP1*. S13 and S21 are missense variations in the *BRCT domain-containing protein* gene and the *Guanine nucleotide-binding protein* gene, respectively. S3 and S9 are located in a *Dynamin family protein* gene, representing a missense variation and an extra stop codon, respectively. S16 and S19 are missense variations in *CINNAMYL ALCOHOL DEHYDROGENASE 4*. Finally, S1, which is significant only in the *indica* group, is a missense variation in a *hypothetical protein* gene.

**Table 3:** Information about significant SNP loci and PHS and GI distribution by genotype in 80 *japonica* and 64 *indica* accessions.

Ecotype	Group	Name	Loci	Type	*N*	*F*-value	PHS	*F*-value	GI
*japonica*	Genotype	*qLTG3-1* ^27,37^	Chr3 (220067-220621)	normal	67	7.01^∗∗^	38.4 ± 26.1 b	15.83^∗∗^	8.6 ± 4.7 b
				72 bp del	13		16.1 ± 23.4 a		3.2 ± 2.7 a
		V2	Chr11_27656171	AA	49	10.66^∗∗^	42.3 ± 28.4 a	8.225^∗^	8.9 ± 5 a
			(OS11G0683500, *Os11bglu36*)	GG	31		23 ± 18.5 b		5.8 ± 3.9 b
		S4	Chr1_39719385	CC	73	6.071^∗^	37.1 ± 26.6 a	5.268^∗^	8.1 ± 4.8 a
			(OS01G0911700, *OsVP1*)	GG	7		10.4 ± 11.2 b		3.8 ± 2.8 b
		V5	Chr7_4576008	CC	26	5.713^∗^	44.4 ± 27.9 a	2.225	8.8 ± 4.2
			(OS07G0186000, *OsTRXh1*)	AA	54		30.2 ± 24.8 b		7.1 ± 5
		S13	Chr1_41233179	CC	55	5.9^∗^	29.9 ± 25.3 b	3.446	7 ± 5
			(OS01G0939300,	TT	25		45.6 ± 26.5 a		9.2 ± 4.1
			*BRCT domain containing protein*)						
		S21	Chr6_637769	GG	72	4.414^∗^	32.8 ± 25.6 b	0.002	7.7 ± 4.9
			(OS06G0111400, G-protein)	AA	8		52.9 ± 29.8 a		7.6 ± 3.5
		S3, S9	Chr3_8434986, Chr3_8435901	CC	27	1.416	27.4 ± 28	4.406^∗^	6 ± 4.3 ab
			(OS03G0260000,	CT	1		26.3 ± 0		0.3 ± 0 b
			*Dynamin family protein*)	TT	52		38.8 ± 25.4		8.7 ± 4.7 a
		S16, S19	Chr11_24303075,	TT	54	1.715	32.2 ± 25.4	8.483^∗∗^	6.6 ± 4.3 b
			Chr11_24307321	AA	26		40.2 ± 28.6		9.9 ± 5.2 a
			(OS11G0622800, *OsCAD4*)						
	Phenotype		Awn	None	44	4.759^∗^	29.1 ± 24.5 b	6.103^∗^	6.5 ± 4.4 b
				Present	36		41.8 ± 27.6 a		9.1 ± 4.9 a

*indica*	Genotype	S1	Chr1_3283359	GG	39	10.67^∗∗^	15 ± 22.3 b	8.894^∗∗^	4 ± 4.3 b
			(OS01G0162900,	GC	18		24.2 ± 30.1 b		5.3 ± 5.5 b
			*Hypothetical protein*)	CC	7		63 ± 25.1 a		12.5 ± 5.1 a
		S13	Chr1_41233179	CC	63	5.42^∗^	21.9 ± 28.1 b	4.413^∗^	5.1 ± 5.3 b
			(OS01G0939300,	TT	1		84.5 ± 0 a		16.4 ± 0 a
			*BRCT domain containing protein*)						
		V2	Chr11_27656171	AA	60	3.14	21.2 ± 28.1	4.81^∗^	4.9 ± 5.3 b
			(OS11G0683500, *Os11bglu36*)	GG	4		48.3 ± 30.6		11 ± 3.8 a

*P-value: ^∗∗^ and ^∗^ indicate significance at the 1 and 5% level, respectively.*

Seven new PHS- and GI-associated loci (V2, S4, V5, S13, S21, S16, and S19) in the *japonica* group are not included among previously reported QTLs, whereas the *Dynamin family protein* gene (OS03G0260000), containing the S3 and S9 variations associated with GI, overlaps with reported QTLs including *Sdr1* (Takeuchi et al., 2003), *qDT-SGC3.1* (Jiang et al., 2011) and *qSD-3* (Guo et al., 2004). Among the three loci that are significantly associated with PHS in the *indica* group, S1, located on the short arm of chromosome-1, overlaps with reported QTLs including *Sdr6* (Marzougui et al., 2012), *qSD-1* (Miura et al., 2002), *qSD1* (Gu et al., 2004, 2006), *qDEG1* (Li et al., 2011). We investigated the spatio-temporal expression patterns of genes harboring significant PHS- and GI-associated SNPs by surveying a publically available expression database (RiceXPro).^4^ *OsVP1* (S4 variation), *BRCT domain containing protein* (S13 variation), and *Hypothetical protein* (S1 variation) are highly expressed in embryos and endosperm compared with other tissues, whereas *G-protein* (S21 variation) is expressed at low levels in embryos and endosperm during seed maturation (data not shown). *Dynamin family protein* (S3 and S9 variation), *THIOREDOXIN H-TYPE* (V5 variation), and *OsCAD4* (S16 and S19 variation) are more highly expressed in endosperm than in embryo tissue, while *BETA-GLUCOSIDASE 36* (V2 variation) is expressed at lower levels in endosperm than in embryo tissue.

### Development and Validation of a Regression Model for PHS in *japonica* Rice

We conducted regression analysis based on the genotypes of 11 significant loci including *qLTG3-1* and PHS values among 80 *japonica* accessions. The genotypes of significant loci and the presence/absence of awns were converted into binary data (Supplementary Table 9): the reference genotype was coded as “0,” while alternative types were coded as “1”; the absence of awns was coded as “0,” and their presence was coded as “1.” We performed regression analysis using this data set and calculated parameter estimates, *p*-values, and adjusted *R^2^* values accordingly. The regression model revealing the highest adjusted *R^2^* value was adopted for PHS estimation, with an adjusted *R^2^* value of 0.401 [PHS predicted value = 33.082 - 12.171(qLTG3.1) - 14.479(V2) - 11.629(V5) - 20.62(S4) + 22.544(S21) + 12.209(S3) + 10.864(S13) + 11.767(Awn)] (**Table 4**). The regression model is composed of eight factors, including six SNP loci (five loci – significant in PHS, one locus – significant in GI), one InDel locus of *qLTG3-1*, and the awn character.

**Table 4:** Regression equation for PHS in *japonica*.

Name	Loci	Parameter estimate	*P*-value
qLTG3-1	qLTG3-1	–12.171	^∗^
V2	Chr11_27656171	–14.479	^∗∗∗^
V5	Chr7_4576008	–11.629	^∗∗^
S4	Chr_1_39719385	–20.62	^∗∗^
S21	Chr_6_637769	22.544	^∗∗^
S3	Chr_3_8434986	12.209	^∗∗^
S13	Chr_1_41233179	10.864	^∗∗^
Awn		11.767	^∗^
Intercept		33.082	^∗∗∗^
Adjusted *R^2^*		0.401	

*P-value: ^∗∗∗, ∗∗^, and ^∗^ indicate significance at the 1%, 5%, and 10% level, respectively.*

**FIGURE 2:**
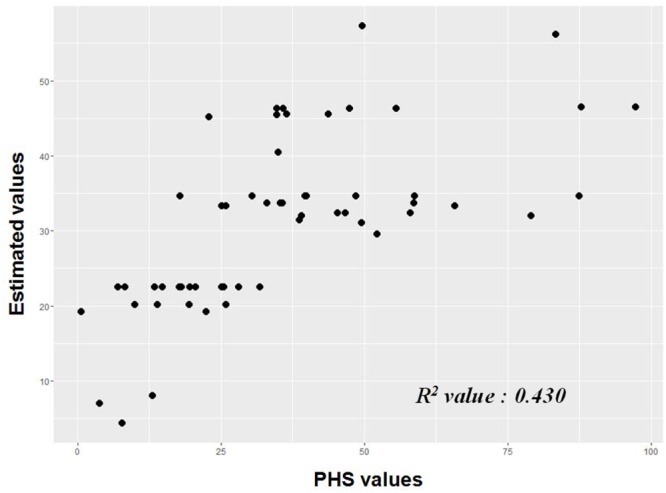
Validation of regression equation model for PHS; 56 *japonica* accessions. ^∗^ [PHS predicted value = 33.082 - 12.171(qLTG3.1) - 14.479(V2) - 11.629(V5) - 20.62(S4) + 22.544(S21) + 12.209(S3) + 10.864(S13) + 11.767(Awn)]

To validate the estimation model for PHS, we genotyped 56 additional Korean *japonica* accessions and measured the PHS value at 42 DAF (Supplementary Table 10). The estimated PHS values derived from the PHS regression equation were calculated and compared to actual PHS measurements (**Figure 2**). The *R^2^* (coefficient of determination) value between the predicted PHS estimates and the actual measurements was 0.430 (*p-values* < 0.001).

## Discussion

The regulation of PHS has become a crucial issue due to global climate change, as unpredictable moisture conditions can lead to PHS, which reduces both the quality and quantity of usable grain. Several studies investigated the molecular mechanism underlying PHS caused by the breakage of adequate dormancy, including the identification and fine-mapping of QTLs related to seed dormancy and PHS (Hattori et al., 1994; Bentsink et al., 2006; Fang et al., 2008; Fujino et al., 2008; Gu et al., 2010; Sugimoto et al., 2010; Gu et al., 2011; De Laethauwer et al., 2012). While GWAS using 350 worldwide accessions revealed germination associated 16 loci (Magwa et al., 2016), most studies investigating PHS and seed dormancy in rice have been limited to a few genetic resources. Therefore, in the current study, we surveyed naturally occurring alleles in rice, a model cereal crop, to expand the available alleles for PHS based on phenotypic variations in PHS among diverse rice genetic resources (Lee et al., 2016).

We conducted enrichment analysis using Fisher’s exact test to identify differential SNPs between PHS resistant and susceptible accessions to find effective genic SNPs while a linear or mixed model generally have been used for GWAS in plant population (Korte and Farlow, 2013). These SNPs, which reveal increased allelic frequency in either PHS resistant or susceptible rice, might be associated with PHS. While the loss of positive loci or the inclusion of negative loci for PHS might be possible due to the small analyzed samples (21 accessions), these loci could serve as the basis for finding candidate genes affecting the quantitative trait, PHS. We detected approximately 21,000 and 18,000 SNPs revealing distinct allelic distribution between PHS resistant and susceptible accessions (*p-value* < 0.05), respectively, including approximately 4,100 and 3,700 variations in genic regions based on dominant and recessive models, respectively. We constructed a density plot of SNPs showing allelic differentiation between PHS resistant and susceptible accessions, finding different densities in different chromosomal regions. Some regions containing high numbers of these SNPs overlap with previously reported QTLs associated with PHS and seed dormancy; Region-1 and Region-2 of Chromosome-1, Region-3 of Chromosome-3, Region-6 of Chromosome-7, and Region-7 of Chromosome-12 overlap with previously reported QTLs and fine-mapped genes (Miura et al., 2002; Dong et al., 2003; Takeuchi et al., 2003; Gu et al., 2004, 2006, 2010; Sugimoto et al., 2010; Jiang et al., 2011; Li et al., 2011; Lu et al., 2011; Marzougui et al., 2012). The most genome-wide germination associated loci reported by GWAS in rice germplasm (Magwa et al., 2016) were included in the regions revealing high number of differential SNPs between PHS resistant and susceptible accessions. In this regard, these chromosomal regions might include several genes that are strongly associated with PHS, which could serve as the basis for finding valuable alleles for PHS regulation.

Weedy rice is characterized by seed shattering, higher dormancy, and the presence of awns compared with cultivated rice (Oka, 1988; Cao et al., 2006). Gu et al. (2003) reported that the presence of an awn was negatively correlated with seed germination in F_2_ populations derived from a weedy rice strain. In the current study, although the presence of an awn was not significantly correlated with PHS and GI in the *indica* accessions examined, *japonica* accessions with awns showed higher PHS and GI than those lacking awns. Therefore, the presence of an awn might be associated PHS in domesticated *japonica* accessions, which might reflect the different genetic constitutions between *japonica* and *indica* ecotype (Morishima and Oka, 1981; Sun et al., 2002; Sun et al., 2015). We ultimately acquired six and seven SNP loci that were significantly associated with PHS and GI, respectively, in *japonica* rice. Among these loci, the S3 and S9 loci in the *Dynamin family protein* gene (OS03G0260000) are included in the chromosomal region harboring germination-related QTLs such as *Sdr1* (Takeuchi et al., 2003) *Hd8* (QTL for heading date), *qDT-SGC3.1* (Jiang et al., 2011), and *qSD-3* (Guo et al., 2004). S1, which is significant only in *indica* rice, is a variant in the *Hypothetical protein* gene (OS01G0162900). This locus is included in the chromosomal region of germination-related QTLs *Sdr6* (Marzougui et al., 2012), *qSD-1* (Miura et al., 2002), *qSD1* (Gu et al., 2004, 2006), and *qDEG1* (Li et al., 2011). Genes containing these significant SNP loci and overlapping with reported QTL regions might be functional genes for the regulation of PHS. The other genes possessing significant SNP loci are not located in reported major QTL regions. However, these genes might be associated with PHS, as these loci might represent new alleles in natural populations that were difficult to detect in previous general QTL studies using restricted sets of parents.

Using the genotypes of significant SNP loci, we constructed a regression equation for PHS in *japonica* rice. The regression equation showing the highest adjusted *R^2^* value (0.401) included seven genetic factors and one phenotypic factor (absence or presence of an awn). We validated the regression equation model, finding that the *R^2^* value between the predicted and actual values was 0.430 (*p-values* < 0.001). As PHS is a quantitative trait that is regulated by complex factors, it is reasonable to expect that the adjusted *R^2^* value and the correlation between actual and predicted values would be low.

Several QTLs and significant SNPs have been reported for seed dormancy and PHS, and finding additional factors associated with PHS might increase the resolving power of our equation. Based on this study, we will try to elaborate the regression equation for PHS including previously reported high quality SNPs, and this might be valuable for the molecular breeding for PHS resistance.

## Author Contributions

G-AL: performed research, analyzed data, wrote the paper. Y-AJ: phenotyped samples, analyzed data. H-SL: performed research, wrote the paper. DH: performed research, analyzed data. J-RL: performed research, analyzed data. M-CL: performed research, analyzed data. S-YL: designed research, performed research. K-HM: designed research, performed research. H-JK: designed research, performed research, wrote the paper.

## Conflict of Interest Statement

The authors declare that the research was conducted in the absence of any commercial or financial relationships that could be construed as a potential conflict of interest.

## Footnotes

1 http://picard.sourceforge.net

2 http://www.r-project.org/

3 http://ricexpro.dna.affrc.go.jp

4 http://ricexpro.dna.affrc.go.jp/
